# Phenotyping the obesities: reality or utopia?

**DOI:** 10.1007/s11154-023-09829-x

**Published:** 2023-08-04

**Authors:** Piero Portincasa, Gema Frühbeck

**Affiliations:** 1 Department of Precision and Regenerative Medicine and Ionian Area (DiMePre-J), Clinica Medica “A. Murri”, University of Bari Medical School, 70124 Bari, Italy; 2 Department of Endocrinology and Nutrition, Clínica Universidad de Navarra, 31008 Pamplona, Spain; 3 Metabolic Research Laboratory, Clínica Universidad de Navarra, 31008 Pamplona, Spain; 4 Centro de Investigación Biomédica en Red Fisiopatología de La Obesidad Y Nutrición (CIBEROBN), 31008 Pamplona, Spain; 5 Navarra Institute for Health Research, IdiSNA, 31008 Pamplona, Spain

**Keywords:** Obesity, Phenotypes, Comorbidities, Heterogeneity, Patient stratification, Clustering

## Abstract

In this thematic issue on phenotyping the obesities, prominent international experts offer an insightful and comprehensive collection of articles covering the current knowledge in the field. In order to actually capture all the polyhedral determinants of the diverse types of obesity, the granularity of the phenotypic information acquired must be expanded in the context of a personalized approach. Whilst the use of precision medicine has been successfully implemented in areas like cancer and other diseases, health care providers are more reluctant to embrace detailed phenotyping to guide diagnosis, treatment and prevention in obesity. Given its multiple complex layers, phenotyping necessarily needs to go beyond the multi-omics approach and incorporate all the diverse spheres that conform the reality of people living with obesity. Potential barriers, difficulties, roadblocks and opportunities together with their interaction in a syndemic context are analyzed. Plausible lacunae are also highlighted in addition to pointing to the need of redefining new conceptual frameworks. Therefore, this extraordinary collection of state-ofthe-art reviews provides useful information to both experienced clinicians and trainees as well as academics to steer clinical practice and research in the management of people living with obesity irrespective of practice setting or career stage.

The obesity landscape has evolved enormously in the past years with its interest and the pace of discovery advancing at a phenomenal speed [1, 2]. Nonetheless, during the last decades the obesity pandemic has gained even more momentum worldwide and is expected to continue increasing unless properly understood and addressed. This is notoriously relevant especially regarding its translation into elevated psychosocial burden, morbidity, premature death, and economic impact [3].

Obesity is currently defined as an “excess or abnormal fat accumulation that may impair health” [4]. In addition, obesity is a complex, heterogeneous, chronic, relapsing disease state that entails multidimensional aspects as regards its presentation, pathophysiology, progression, and response to treatment. Therefore, it is more appropriate to use the term “obesities” in plural [5]. In order to actually capture all the polyhedral determinants of the disease, the granularity of the phenotypic information acquired must be expanded in the context of a personalized approach. However, while the use of precision medicine applying detailed phenotyping to guide diagnosis, treatment and prevention has been successfully implemented in areas like cancer and other diseases, health care providers are more reluctant to embrace this approach in the field of obesity. We believe this is still an unmet need.

For this reason we are hugely honored to serve as *Guest Editors* of this special issue on ***“Phenotyping the obesities”***. Together with the contributions of worldwide experts in the field, we aim to provide readers an exhaustive and comprehensive insight into the current state of knowledge. Noteworthy, when considering obesity, every person should be assessed based on their own specific and unique circumstances. In this line, Trang and Grant [6] review the contribution of both genetic and epigenetic factors to obesity susceptibility. Among others, they analyze key signatures in obesity development and delineate epigenetic changes potentially underlying environmental modulation.

Formerly regarded as a monotonous, passive and unattractive organ, adipose tissue has emerged as a highly communicative, versatile and dynamic tissue, as well as a very active source of secretory factors with a broad range of subsequent organ alterations and regulatory implications that lie at the heart of the ABCD concept of obesity, i.e. an adiposity-based chronic disease [7, 8]. Chronic low-grade inflammation, insulin resistance, and oxidative stress are some of the hallmarks that characterize adiposity-related disease with its accompanying lipotoxicity translating into organ damage. Therefore, people living with obesity (PlwO) should undergo a holistic diagnostic approach that contemplates a thorough and exhaustive evaluation of comorbidities.

In the context of precision medicine, Perdomo et al. [9] put forward an innovative patient-centered framework. The underlying mechanistic links between adiposity and obesity-associated comorbidities will continue to represent a fertile research area. While the list of currently known adipose-derived molecules is extensive [10–20], others are most probably still awaiting to be described. Characterising these and exploring their effects will certainly provide additional underpinnings explaining how dysfunctional fat excess fosters the development of comorbidities and underlies adiposopathy. In this scenario, detailed body composition analyses emerge as indispensable determinations to fully capture the amount, distribution and proportion of adiposity and fat-free mass [21]. Whilst body mass index (BMI) remains the most frequently used classification approach for PlwO, it does not precisely reflect total body adiposity as well as its distribution. Salmón-Gómez et al. [21] outline both classical procedures and a new classification system for phenotyping the obesities underscoring the pertinence of incorporating them into the clinical setting on a regular basis.

Noteworthy, obesity does not spare any organ with a predilection for metabolic derangements and low-grade chronic inflammation. Not surprisingly, the epidemiology of several diseases has been already influenced by obesity. For instance, non-alcoholic fatty liver disease (NAFLD) or its progressive form, non-alcoholic steatohepatitis (NASH), have long been recognised in association with excess adiposity, and such a close “metabolic” link has merited the attention of experts worldwide with a proposed shift in terminology [22, 23]. Moreover, as described by Francque [24] the unravelling of the mechanistic understanding is hampered by a huge hepatic heterogeneity as regards coping and repair systems dealing with inflammation and metabolic stress. The existing ample inter-individual variability also relies on genetic and epigenetic influences that could aid in stratification of risk albeit not fully explaining the huge disease heterogeneity. Hepatic tissue analyses together with non-invasive procedures will certainly contribute to gain more insight into the pathology and response heterogeneity. Tailoring of currently available dietary interventions [25] based on liver phenotypes as well as druggable pathology-driving pathways can be foreseen in the not so distant future. The heart represents a further excellent example of how obesity affects all organs. Excess weight causes not only structural and functional cardiovascular alterations but also hemodynamic and humoral ones. Deep phenotyping is required to adequately and accurately adscribe the diverse obesity phenotypes to cardiometabolic risk at the same time as guiding in the selection of the best therapeutic approach for its clinical management as discussed by Preda et al. [26]. The appreciation of the relevance of pathophysiological organ crosstalk triggered by increasingly applied computational techniques aimed at data integration and interdisciplinary knowledge of multiorgan crosstalk reveal novel opportunities (Fig. 1).

**Fig. 1:**
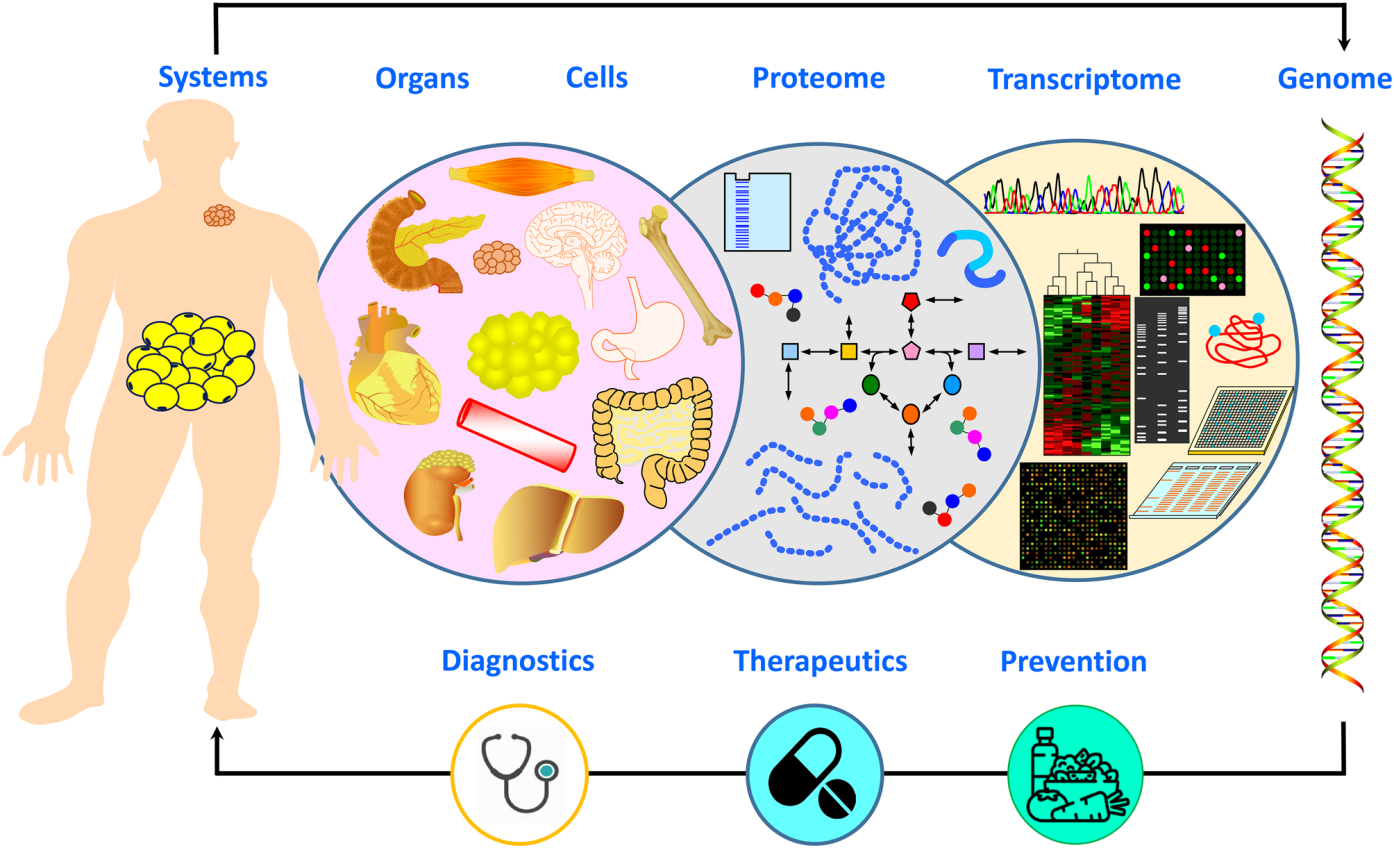
Holistic systems biology approach aimed at data integration and interdisciplinary knowledge of multiorgan crosstalk reveal novel opportunities for obesity phenotyping

Blaak and Goossens [27] review the existence of diverse metabolic phenotypes or metabotypes in the context of obesity. Moreover, insulin resistant metabotypes are dissected, highlighting the different etiologies towards cardiovascular disease and type 2 diabetes (T2D). Interestingly, it is shown that the described tissue insulin resistant metabotypes behave differentially in response to diet. Therefore, efficient management strategies for PlwO should target the described metabotypes for potentially better cardiometabolic health outcomes. Thus, the implementation of precision nutrition approaches according to obesity metabotypes should be pursued irrespective of effects on weight loss. Relevant drivers of the response to dietary interventions include age, sex, microbial composition and functionality. A complex polymicrobial ecology of about 100 trillion (10^14^) microorganisms exists in the human gut at the interface of internal and external environment over a surface of roughly 200–300 m^2^. The two major *phyla* in the human gut are the *Firmicutes* (60%) which are enriched in Gram-positive bacteria, and the *Bacteroidetes* (10%) which consist of Gram-negative anaerobic bacteria. Given the current knowledge of the relevance of the microbiota in metabolism control and energy homeostasis, Di Ciaula et al. [28] argue that a comprehensive phenotyping of PlwO should incorporate the analysis of its composition, potential shifts towards dysbiosis, bacterial products including secondary bile acids, metabolite secretion, receptor activity as well as overall contribution and effect on gut barrier integrity, among others.

In addition to the biological phenotyping, other equally relevant spheres need to be contemplated and can not be dismissed. In this line, Camacho-Barcia et al. [29] put forward that the psycho-behavioural characterization of PlwO better captures the powerful influences or determinants of human feeding behaviour. Characteristic psycho-behavioural features potentially contributing to difficulties in body weight maintenance and loss include cognitive control, reward dependence, as well as mood and emotion. Interestingly, a notable overlapping of the psycho-behavioural phenotypes takes place, thereby hinting to the plausible bidirectional interplay between these traits. The stratification and identification of these profiles as intervention targets may lead to an improved treatment response in PlwO. Undoubtedly, focusing on the intricacies of the human reward system will yield more applicable information to everyday practice than endlessly excavating in the appetite control pathways of rodents.

Obesity management still remains among the spiniest pathologies to treat as well as one of the greatest unmet needs. A deep view at why certain approaches succeed or fail is needed given the huge interindividual variability in response to all types of treatment [2]. Traditionally dietary intervention paradigms have not considered the heterogeneity in pathophysiology as well as in treatment response. Shannon et al. [30] address the diversity in physiological and postprandial phenotypes underscoring how precision nutrition can translate into tailored, more efficient approaches with improved cardiometabolic outcomes beyond simple weight loss. Similarly, the diverse types of exercise exert differential effects with increases in cardiorespiratory fitness being observed following aerobic as well as combined aerobic and resistance training, while amelioration in muscle strength is triggered by resistance exercise, but not after aerobic training [31]. Whilst physical activity has a moderate effect on body weight, its overall benefits should not be underestimated with long-term adherence remaining a big challenge. In this context, phenotype-tailored lifestyle interventions reportedly attain important weight loss, which will need confirmation of causality in apropriate trials [32].

Anti-obesity pharmacotherapy currently encompasses several types of drugs that are adequately effective and acceptably safe thereby having reached clinical practice. Hocking and Sumithran [33] highlight that whilst appealing, treatment individualization to patient characteristics is not routinely performed in the clinical setting besides in rare cases of monogenic obesity. Deep phenotyping aided by advances in technology will sophisticate drug prescription in the future. So far, however, only early weight loss is related to long-term weight loss but is not useful to guide treatment choice when starting medication. Some proof-of-concept studies towards tailored and targeted approaches have already attempted to differentiate distinct phenotypes of obesity that could prevent the development of cardiometabolic diseases [34] or predict treatment response to anti-obesity drugs [35, 36].

While some factors of success following bariatric surgery (BS) like age, gender, genetic, neuroendocrine and metabolic features, comorbidity history as well as psychosocial and economic circumstances determine outcomes, none can completely and robustly predict the specific patient response [37]. Although BS techniques are safe in experienced hands and teams, several long-term complications can arise. Among them nutritional deficiencies stand out [38]. Therefore, focusing attention on dietary recommendations not only after BS but also preoperatively is a necessity. Undoubtedly, precision nutrition approaches will optimize BS outcomes and decrease potential harms. Surgery-induced weight loss varies individually. Underlying mechanisms driving individual differences in weight loss and regain include type of surgery, eating behaviour and genetics, among others. Cohen and Petry [39] describe the comprehensive and individual evaluation that patients should undergo to address in a personalized way insufficient weight loss or weight regain suggesting a customized algorithm. While conversion and revision surgeries can be contemplated, current pharmacological options provide a useful adjunctive. It is tantalising and not that far-fetched to speculate that artificial intelligence will help in the application of prediction algorithms in a precision medicine scenario.

Surprisingly, precision public health has not yet being firmly established and represents an evolving concept. Baker and Bjerregaard [40] view data access and completeness together with its integration, universal inclusion of society members, ethical issues, and final translation into true policy measures as arising challenges. As opposed to sterile public health initiatives in the past decades, precision public health proposals may produce new advances fostering active policies that could ultimately lead to more useful prevention of childhood obesity. In this line, identification of children with already metabolic alterations associated to obesity should be absolutely a clinical priority [41, 42].

Unlike other fields in which phenotyping focuses only on omics approaches, in obesity given its multiple complex layers, phenotyping necessarily needs to go beyond the multi-omics approach and incorporate capturing all the diverse spheres that conform PlwO. In this sense, with a particular emphasis on a detailed anamnesis, inclusion of a thorough physical examination and comorbidity assessment that includes the psychological as well as the socioeconomic circumstances that shape the potential barriers, roadblocks and opportunities is required together with their interaction in a syndemic context [43] as depicted in Fig. 2.

**Fig. 2:**
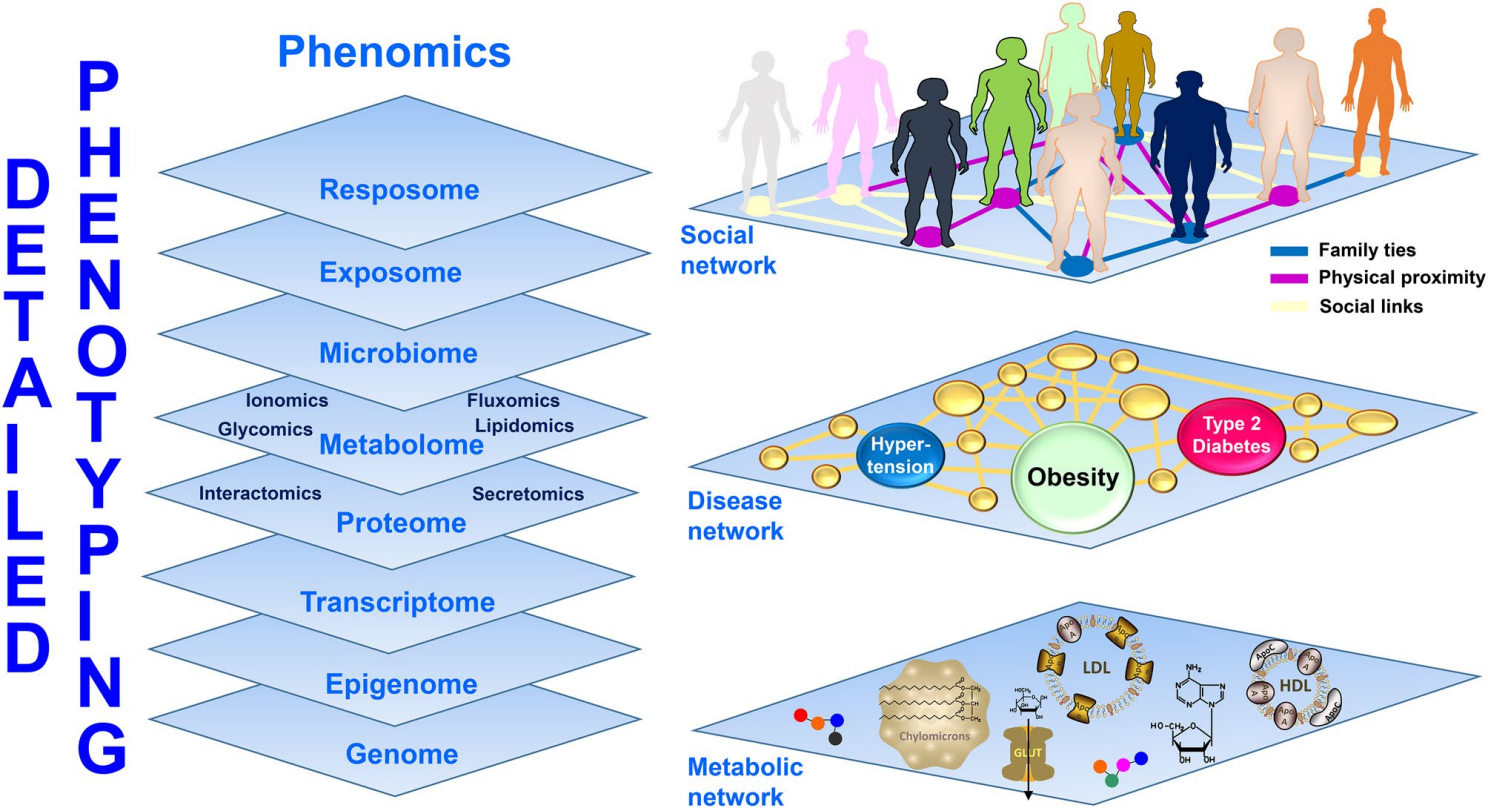
Multi-omics phenotyping and network interaction in a syndemic context

The precise diagnosis and follow-up of obesity will depend on the granularity of the determinations that we believe need to be acquired to fully comprehend all obesity manifestations in a holistic way. The ongoing scenario is more complex than it was once believed to be. The overwhelming impression is the existence of multiple systems coming into play under particular circumstances to shape the appearance of the diverse obesities, rather than a simplistic top-down hierarchy. A frequent observation that recurs throughout the different reviews is the difficulties in better phenotyping PlwO. The plausible lacunae are also highlighted in addition to speculating on prospective changes with the opportunity of redefining new insights. Remaining questions pertain to whether detailed phenotyping will effectively outstrip current practice at the same time as improving its management, overall health and societal hazards. A further aspect to be considered is the cost-effectiveness of implementing an array of determinations and how they will translate into better diagnosis, treatment, prevention and well being at large. Given the refractoriness of obesity, it is important not to left it untreated. Detailed phenotyping places us to better explore the physiological, psychological and perhaps even the molecular genetic determinants of treatment compliance and outcome, but will need a large-scale, dispassionate and coordinated approach for its corroboration, a combination that can prove elusive.

When dealing with obesity, the elaboration of an actionable and targeted plan to address its potential causes, barriers as well as successful management is of utmost importance. While these are exciting times for obesity, the next decade will be critical for applying and consolidating the knowledge gained, and particularly for challenging our collective ability to properly diagnose and manage this disease as well as adequately address all its accompanying circumstances. In the not so distant future, it can be envisaged that direct insight into the pathophysiological mechanisms of obesities and their phenotypical expression may be gained at a fingertip (Fig. 3). A phenotype-based approach framework targeting specific pathways will allow for more effective and sustained therapeutic impact in PlwO. Leveraging artificial intelligence with clinician validation will enable task-oriented follow-up and proactive outreach with a patient-centered, sustainable and scalable approach [44].

**Fig. 3:**
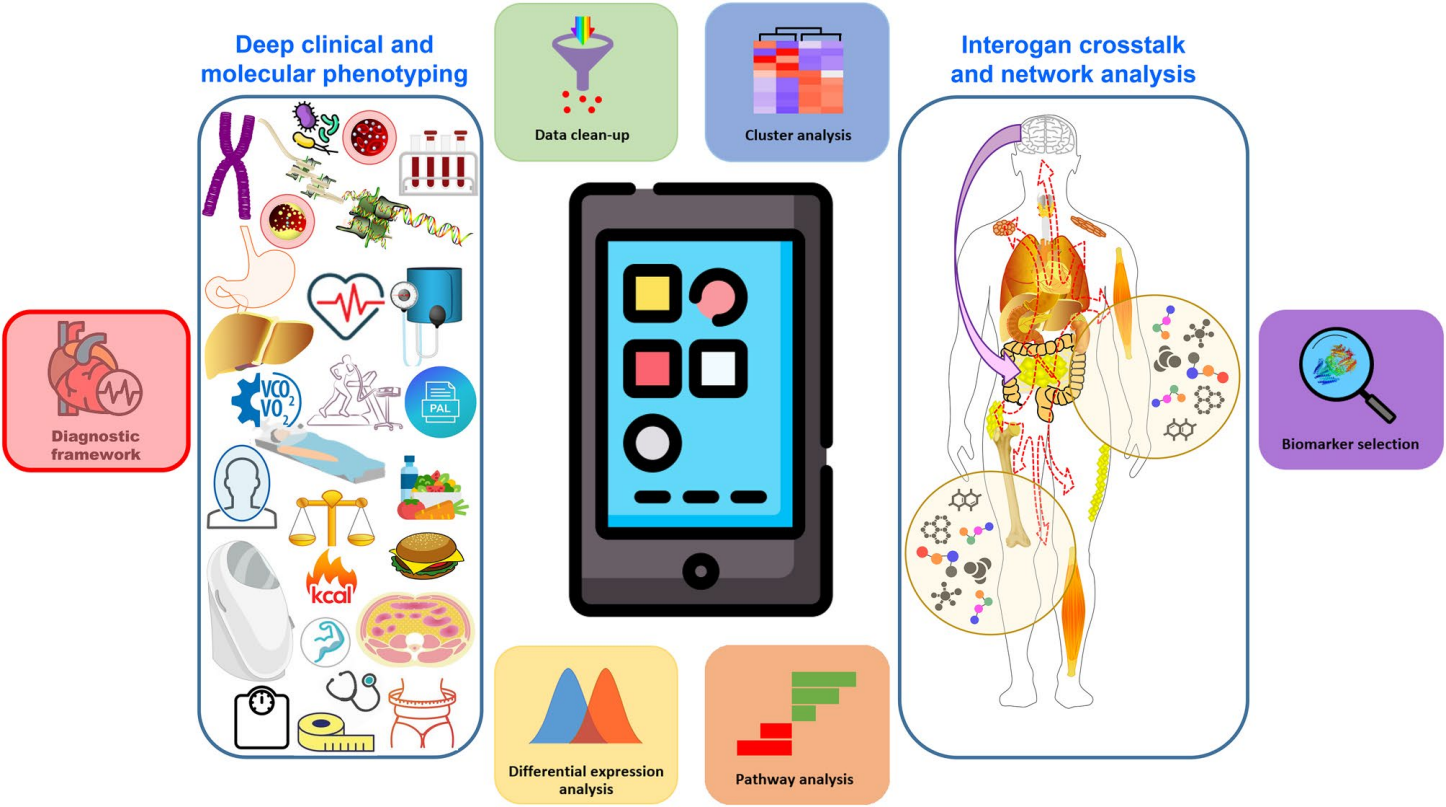
Future framework for phenotyping the obesities

## References

[1] Frühbeck G, Gómez-Ambrosi J (2003). Control of body weight: a physiologic and transgenic perspective. Diabetologia.

[2] Perdomo CM, Cohen RV, Sumithran P, Clément K, Frühbeck G (2023). Contemporary medical, device, and surgical therapies for obesity in adults. Lancet.

[3] Catalán V, Avilés-Olmos I, Rodríguez A, Becerril S, Fernández-Formoso JA, Kiortsis D (2022). Time to consider the “exposome hypothesis” in the development of the obesity pandemic. Nutrients.

[4] World Health Organization. Fact sheet: obesity and overweight. Available at: http://www.who.int/mediacentre/factsheets/fs311/en/. Updated 9 June 2021.

[5] Yárnoz-Esquiroz P, Olazarán L, Aguas-Ayesa M, Perdomo CM, García-Goñi M, Silva C, et al. 'Obesities': position statement on a complex disease entity with multifaceted drivers. Eur J Clin Invest. 2022;e13811. 10.1111/eci.13811 PMC9285368 35514242

[6] Trang K, Grant SFA. Genetics and epigenetics in the obesity phenotyping scenario. Rev Endocr Metab Disord. 2023.10.1007/s11154-023-09804-6. (Epub ahead of print Apr 10:1–19):PMID: 37032403. 10.1007/s11154-023-09804-6 PMC10088729 37032403

[7] Frühbeck G, Busetto L, Dicker D, Yumuk V, Goossens GH, Hebebrand J (2019). The ABCD of obesity: An EASO Position Statement on a diagnostic term with clinical and scientific implications. Obes Facts.

[8] Sattar N, McMurray JJV, McInnes IB, Aroda VR, Lean MEJ (2023). Treating chronic diseases without tackling excess adiposity promotes multimorbidity. Lancet Diabetes Endocrinol.

[9] Perdomo CM, Avilés-Olmos I, Dicker D, Frühbeck G. Towards an adiposity-related disease framework for the diagnosis and management of obesities. Rev Endocr Metab Disord. 2023. 10.1007/s11154-023-09797-2. (Epub ahead of print May 10):PMID: 37162651. 10.1007/s11154-023-09797-2 PMC10492748 37162651

[10] Frühbeck G, Gómez AJ (2001). Rationale for the existence of additional adipostatic hormones. FASEB J.

[11] Frühbeck G, Gómez Ambrosi J, Salvador J (2001). Leptin-induced lipolysis opposes the tonic inhibition of endogenous adenosine in white adipocytes. FASEB J.

[12] Frühbeck G, Gómez-Ambrosi J (2001). Modulation of the leptin-induced white adipose tissue lipolysis by nitric oxide. Cell Signal.

[13] Muruzábal FJ, Frühbeck G, Gómez-Ambrosi J, Archanco M, Burrell MA (2002). Immunocytochemical detection of leptin in non-mammalian vertebrate stomach. Gen Comp Endocrinol.

[14] Frühbeck G (2005). Obesity: Aquaporin enters the picture. Nature.

[15] Sabater M, Moreno-Navarrete JM, Ortega FJ, Pardo G, Salvador J, Ricart W (2010). Circulating pigment epithelium-derived factor levels are associated with insulin resistance and decrease after weight loss. J Clin Endocrinol Metab.

[16] Catalán V, Gómez-Ambrosi J, Rodríguez A, Ramírez B, Rotellar F, Valentí V (2011). Increased levels of calprotectin in obesity are related to macrophage content: impact on inflammation and effect of weight loss. Mol Med.

[17] Pulido MR, Diaz-Ruiz A, Jimenez-Gomez Y, Garcia-Navarro S, Gracia-Navarro F, Tinahones F (2011). Rab18 dynamics in adipocytes in relation to lipogenesis, lipolysis and obesity. PLoS ONE.

[18] Rodríguez A, Gómez-Ambrosi J, Catalán V, Rotellar F, Valentí V, Silva C (2012). The ghrelin O-acyltransferase-ghrelin system reduces TNF-a-induced apoptosis and autophagy in human visceral adipocytes. Diabetologia.

[19] Moreno-Navarrete JM, Escote X, Ortega F, Serino M, Campbell M, Michalski MC (2013). A role for adipocyte-derived lipopolysaccharide-binding protein in inflammation- and obesity-associated adipose tissue dysfunction. Diabetologia.

[20] Catalán V, Gómez-Ambrosi J, Rodrïguez A, Pérez-Hernández AI, Gurbindo J, Ramírez B (2014). Activation of noncanonical Wnt signaling through WNT5A in visceral adipose tissue of obese subjects is related to inflammation. J Clin Endocrinol Metab.

[21] Salmón-Gómez L, Catalán V, Frühbeck G, Gómez-Ambrosi J. Relevance of body composition in phenotyping the obesities. Rev Endocr Metab Disord. 2023. 10.1007/s11154-023-09796-3. (Epub ahead of print Mar 17):PMID: 36928809. 10.1007/s11154-023-09796-3 PMC10492885 36928809

[22] Rinella ME, Lazarus JV, Ratziu V, Francque SM, Sanyal AJ, Kanwal F, et al. NAFLD Nomenclature consensus group. A multi-society Delphi consensus statement on new fatty liver disease nomenclature. Ann Hepatol. 2023;Jun 20(101133). 10.1016/j.aohep.2023.101133. Epub ahead of print.

[23] Portincasa P (2023). NAFLD, MAFLD, and beyond: one or several acronyms for better comprehension and patient care. Intern Emerg Med.

[24] Francque SMA. Towards precision medicine in non-alcoholic fatty liver disease. Rev Endocr Metab Disord. 2023;online. 10.1007/s11154-023-09820-6 37477772

[25] Perdomo CM, Frühbeck G, Escalada J. Impact of nutritional changes on nonalcoholic fatty liver disease. Nutrients. 2019;11(3):677. 10.3390/nu11030677 PMC6470750 30901929

[26] Preda A, Carbone F, Tirandi A, Montecucco F, Liberale L. Obesity phenotypes and cardiovascular risk: from pathophysiology to clinical management. Rev Endocr Metab Disord. 2023. 10.1007/s11154-023-09813-5. (Epub ahead of print):PMID: 37358728. 10.1007/s11154-023-09813-5 PMC10492705 37358728

[27] Blaak EE, Goossens GH. Metabolic phenotyping in people living with obesity: implications for dietary prevention. Rev Endocr Metab Disord. 2023;online. 10.1007/s11154-023-09830-4 PMC10492670 37581871

[28] Di Ciaula A, Bonfrate L, Khalil M, Garruti G, Portincasa P. Contribution of the microbiome for better phenotyping of people living with obesity. Rev Endocr Metab Disord. 2023. 10.1007/s11154-023-09798-1. (Epub ahead of print Apr 29:1–32):PMID: 37119391. 10.1007/s11154-023-09798-1 PMC10148591 37119391

[29] Camacho-Barcia L, Lucas I, Miranda-Olivos R, Jiménez-Murcia S, Fernández-Aranda F. Applying psycho-behavioural phenotyping in obesity characterization. Rev Endocr Metab Disord. 2023. 10.1007/s11154-023-09810-8. (Epub ahead of print):PMID: 37261609. 10.1007/s11154-023-09810-8 PMC10492697 37261609

[30] Shannon CE, Ní Chathail MB, Mullin SM, Meehan A, McGillicuddy FC, Roche HM. Precision nutrition for targeting pathophysiology of cardiometabolic phenotypes. Rev Endocr Metab Disord. 2023. 10.1007/s11154-023-09821-5. (Epub ahead of print):PMID: 37402955. 10.1007/s11154-023-09821-5 PMC10492734 37402955

[31] Oppert JM, Ciangura C, Bellicha A. Physical activity and exercise for weight loss and maintenance in people living with obesity. Rev Endocr Metab Disord. 2023. 10.1007/s11154-023-09805-5. (Epub ahead of print May 5):PMID: 37142892. 10.1007/s11154-023-09805-5 37142892

[32] Cifuentes L, Ghusn W, Feris F, Campos A, Sacoto D, De la Rosa A (2023). Phenotype tailored lifestyle intervention on weight loss and cardiometabolic risk factors in adults with obesity: a single-centre, non-randomised, proof-of-concept study. EClinicalMedicine.

[33] Hocking S, Sumithran P. Individualised prescription of medications for treatment of obesity in adults. Rev Endocr Metab Disord. 2023. 10.1007/s11154-023-09808-2. (Epub ahead of print):PMID: 37202547. 10.1007/s11154-023-09808-2 PMC10492708 37202547

[34] Fagherazzi G, Zhang L, Aguayo G, Pastore J, Goetzinger C, Fischer A (2021). Towards precision cardiometabolic prevention: results from a machine learning, semi-supervised clustering approach in the nationwide population-based ORISCAV-LUX 2 study. Sci Rep.

[35] Acosta A, Camilleri M, Shin A, Vazquez-Roque MI, Iturrino J, Burton D (2015). Quantitative gastrointestinal and psychological traits associated with obesity and response to weight-loss therapy. Gastroenterology.

[36] Lin Z, Feng W, Liu Y, Ma C, Arefan D, Zhou D (2021). Machine learning to identify metabolic subtypes of obesity: a multi-center study. Front Endocrinol.

[37] Pereira SS, Guimarães M, Monteiro MP. Towards precision medicine in bariatric surgery prescription. Rev Endocr Metab Disord. 2023.10.1007/s11154-023-09801-9. (Epub ahead of print May 2):PMID: 37129798. 10.1007/s11154-023-09801-9 PMC10492755 37129798

[38] Aguas-Ayesa M, Yárnoz-Esquíroz P, Olazarán L, Gómez-Ambrosi J, Frühbeck G. Precision nutrition in the context of bariatric surgery. Rev Endocr Metab Disord. 2023. 10.1007/s11154-023-09794-5. (Epub ahead of print Mar 17:1–13):PMID: 36928810. 10.1007/s11154-023-09794-5 PMC10020075 36928810

[39] Cohen RV, Petry TB. How to address weight regain after bariatric surgery in an individualized way. Rev Endocr Metab Disord. 2023.10.1007/s11154-023-09806-4. (Epub ahead of print May 12):PMID: 37171756. 10.1007/s11154-023-09806-4 37171756

[40] Baker JL, Bjerregaard LG. Advancing precision public health for obesity in children. Rev Endocr Metab Disord. 2023.10.1007/s11154-023-09802-8. (Epub ahead of print Apr 14:1–8):PMID: 37055611. 10.1007/s11154-023-09802-8 PMC10101815 37055611

[41] Faienza MF, Wang DQ, Frühbeck G, Garruti G, Portincasa P (2016). The dangerous link between childhood and adulthood predictors of obesity and metabolic syndrome. Intern Emerg Med.

[42] Faienza MF, Chiarito M, Molina-Molina E, Shanmugam H, Lammert F, Krawczyk M (2020). Childhood obesity, cardiovascular and liver health: a growing epidemic with age. World journal of pediatrics : WJP.

[43] Frühbeck G, Kiortsis DN, Catalán V (2018). Precision medicine: diagnosis and management of obesity. Lancet Diabetes Endocrinol.

[44] Haug CJ, Drazen JM (2023). Artificial intelligence and machine learning in clinical medicine, 2023. N Engl J Med.

